# Combining Immune-Related Genes For Delineating the Extracellular Matrix and Predicting Hormone Therapy and Neoadjuvant Chemotherapy Benefits In Breast Cancer

**DOI:** 10.3389/fimmu.2022.888339

**Published:** 2022-07-14

**Authors:** Jianyu Liu, Bo Lei, Xin Yu, Yingpu Li, Yuhan Deng, Guang Yang, Zhigao Li, Tong Liu, Leiguang Ye

**Affiliations:** ^1^ Department of Breast Surgery, Harbin Medical University Cancer Hospital, Harbin, China; ^2^ Department of Oncology, Harbin Medical University, Harbin, China

**Keywords:** immune, extracellular matrix, breast cancer, neoadjuvant, immune infiltration, microenvironment, hormone therapy

## Abstract

Breast cancer (BC) is the most prevalent cancer in women worldwide. A systematic approach to BC treatment, comprising adjuvant and neoadjuvant chemotherapy (NAC), as well as hormone therapy, forms the foundation of the disease’s therapeutic strategy. The extracellular matrix (ECM) is a dynamic network that exerts a robust biological effect on the tumor microenvironment (TME), and it is highly regulated by several immunological components, such as chemokines and cytokines. It has been established that the ECM promotes the development of an immunosuppressive TME. Therefore, while analyzing the ECM of BC, immune-related genes must be considered. In this study, we used bioinformatic approaches to identify the most valuable ECM-related immune genes. We used weighted gene co-expression network analysis to identify the immune-related genes that potentially regulate the ECM and then combined them with the original ECM-related gene set for further analysis. Least absolute shrinkage and selection operator (LASSO) regression and SurvivalRandomForest were used to narrow our ECM-related gene list and establish an ECM index (ECMI) to better delineate the ECM signature. We stratified BC patients into ECMI high and low groups and evaluated their clinical, biological, and genomic characteristics. We found that the ECMI is highly correlated with long-term BC survival. In terms of the biological process, this index is positively associated with the cell cycle, DNA damage repair, and homologous recombination but negatively with processes involved in angiogenesis and epithelial–mesenchymal transition. Furthermore, the tumor mutational burden, copy number variation, and DNA methylation levels were found to be related to the ECMI. In the Metabric cohort, we demonstrated that hormone therapy is more effective in patients with a low ECMI. Additionally, differentially expressed genes from the ECM-related gene list were extracted from patients with a pathologic complete response (pCR) to NAC and with residual disease (RD) to construct a neural network model for predicting the chance of achieving pCR individually. Finally, we performed qRT-PCR to validate our findings and demonstrate the important role of the gene OGN in predicting the pCR rate. In conclusion, delineation of the ECM signature with immune-related genes is anticipated to aid in the prediction of the prognosis of patients with BC and the benefits of hormone therapy and NAC in BC patients.

## Introduction

According to Global Cancer Observatory (GCO) (https://gco.iarc.fr/), breast cancer (BC) is the most prevalent cancer in women and the second leading cause of female cancer deaths globally. Mastectomy, breast-conserving surgery, sentinel lymph node biopsy (SLNB), and adjuvant or neoadjuvant chemotherapy (NAC) have all become standard treatments for BC, and 5-year and 10-year survival rates of BC patients have significantly improved. However, there is still significant room for improvement in terms of preventing recurrence and improving the long-term outcome *via* a more precise hierarchy of patients (1). Oncologists have utilized numerous molecular subtypes based on protein expression [immunohistochemistry (IHC)] to treat patients for a long time. BC can also be categorized into five molecular subtypes using Partitioning Around Medoids (PAM) based on gene expression profiles: Luminal A, Luminal B, and Basal-like (2). There are significant differences in tumor heterogeneity, incidence, risk factors, prognosis, and treatment sensitivity (3). Systematic treatment, including adjuvant, NAC, and hormone therapy (4), is the backbone of the BC therapeutic strategy. Several models have recently been established and validated to predict the efficacy of chemotherapy response in patients with BC. These models, on the other hand, did not take into consideration the extracellular matrix (ECM).

The ECM is made up of hundreds of different proteins, including glycoproteins, collagens, and proteoglycans (5, 6), that surround cells and form a dynamic and intricate molecular network. This network plays a critical role in protumorigenic and antitumorigenic processes (7). Proteomic analysis of the ECM composition performed on xenograft mice exhibits a unique ECM constitution in cancers with a high metastatic potential (8). Researchers have discovered that ECM rigidity is required for normal cells to transform into cancer *via* Yes-associated protein (YAP)/Transcriptional coactivator with PDZ-binding motif (TAZ) mechanotransduction (9). However, in pancreatic ductal adenocarcinoma, the ECM is also a protective factor, as demonstrated by impairing ECM with an anti-lysyloxidase-like 2 (LOXL2) antibody *in vivo*, hence accelerating tumor progression and decreasing overall survival (OS) (10). In essence, both cancer cells and normal cells can contribute to and be influenced by ECM deposition and remodeling (11). The deposition of the ECM may, in turn, contribute to drug delivery (12).

Intriguingly, in BC, a significant similarity between the ECM and the matrix undergoing wound healing or remodeling was discovered (7, 13). This phenomenon occurs when the mammary gland attempts to revert to its original state after pregnancy, resulting in a significant alteration of the ECM, including robust upregulation of fibrillar, collagens, and certain enzymes (14–16).

To date, several studies have investigated the role of ECM in cancer progression and tumor growth, and some researchers have used genes coding for ECM macromolecular to predict cancer survival and the biological process (3, 3, 17, 18). However, as a component of the tumor microenvironment (TME), the ECM highly interacts with other immunologically relevant components such as chemokines and cytokines, which may be an important mechanism for the ECM to influence the biological process of BC (19, 20). Therefore, focusing solely on the molecular properties of ECM alone will overlook its important function in cancer immunity. As a result, taking into account ECM-interacting immune genes is necessary to delineate a comprehensive landscape of the ECM signature. In this study, we aimed to identify the highly ECM-related genes and used them along with the original ECM gene list to establish a model to predict patient survival and the efficacy of hormone therapy and NAC and discuss its clinical implications.

## Materials and Methods

### Breast Cancer Data Collection and Processing

BC samples with complete clinical annotation were obtained from four databases: The Cancer Genome Atlas (TCGA) database, Gene Expression Omnibus (GEO), University of California Santa Cruz (UCSC) Xena platform (18), and Metabric database. For TCGA cohort (1,092 BC samples), RNA sequencing (RNA-seq) data and corresponding clinical information were extracted from TCGA database (http://cancergenome.nih.gov/) and then transformed into transcripts per kilobase million (TPM). In this analysis, 7 GEO microarray cohorts were used, and expression and survival data were retrieved from the GEO database (https://www.ncbi.nlm.nih.gov/geo/) with background adjustment and normalized using the Robust Multi-Array Average (RMA) algorithm. Expression matrices of 2 UCSC sets were retrieved from the UCSC Xena platform (http://xena.ucsc.edu/). We obtained the Metabric data from cBioPortal (http://www.cbioportal.org/). Before further analysis, all gene expression data were log2-transformed and quantile-normalized using the normality between array techniques in the R package limma 3.46.0. We eliminated the batch effects from the analysis when using merged gene expression data from different datasets *via* the R package sva 3.36.0.

### Gene Set Variation Analysis and Weighted Gene Co-Expression Network Analysis Identification of the Extracellular Matrix-Related Immune Genes

The ECM score of the “core matrisome” gene set downloaded from MatrisomeDB (http://www.pepchem.org/matrisomedb) was quantified for each BC sample using the gene set variation analysis (GSVA) algorithm in the R package GSVA 1.36.2 (21). Weighted correlation network analysis [weighted gene co-expression network analysis (WGCNA)] is a systems biology method for identifying correlation patterns among genes across microarray samples. WGCNA was performed using the WGCNA package in R 3.6.1. Gene significance was used to determine the correlation between individual genes and the ECM score, whereas module membership represented the relationship between module eigengenes and gene expression profiles. To ensure a scale-free topology network, a power of β = 3 and a scale-free R2 = 0.9 were set as soft threshold parameters. Following the retrieval of six modules, the Brown module with the most solid relationship was selected for further analysis. Genes within the Brown module were included in the Gene Ontology (GO) and Kyoto Encyclopedia of Genes and Genomes (KEGG) functional enrichment analyses using the R package clusterProfiler 3.18.1. Through Metascape (HTTPS://metascape.org/), we identified all statistically enriched terms (GO/KEGG terms, Canonical pathways, Hallmark gene sets) in the Brown module. Accumulative hypergeometric p-values and enrichment factors were calculated and used for filtering.

### Clinical and Multi-Omics Data Collection

The most recent clinical data for TCGA-BRCA and other cohorts were directly downloaded as attachment files from corresponding databases. Multi-omics data of TCGA-BRCA cohort, including somatic mutation copy number variation (CNV), somatic mutations [single-nucleotide polymorphisms (SNPs) and small insertions and deletions (INDELs), 22ct2 Variant Aggregation and Masking], and somatic CNV, corresponding to the cases with RNA-seq data, were downloaded from Xenahubs (https://figshare.com/articles/dataset/TCGA-BRCA_mutect2_snv_tsv/19948121). In addition, a GISTIC analysis was performed to determine the enrichment of genomic events. CNVs in two clusters and the threshold copy numbers at alteration peaks were obtained using Genomic Identification of Significant Targets in Cancer (GISTIC) 2.0 analysis (https://gatk.broadinstitute.org). DNA methylation (Illumina Human Methylation 450K) was obtained from UCSC Xena (https://xenabrowser.net/).

### Unsupervised Clustering for the Extracellular Matrix Constitution

Using the R package ConsensuClusterPlus 1.54, it was possible to identify robust ECM clusters in TCGA patients using a consensus clustering technique of partition (based on the Euclidean distance and Ward’s linkage) in conjunction with the 11 genes obtained by LASSO regression. The cumulative distribution function (CDF) and consensus heatmap were used to determine the optimal K value. This procedure was repeated a total of 1,000 times to ensure the stability of the stratification process.

### Construction and Validation of the Extracellular Matrix Index

TCGA training set was subjected to univariate Cox regression analysis to identify genes associated with prognosis with a p-value <0.01. To obtain a quantitative description of the survival risk of each patient, LASSO regression analysis was further used to calculate the ECM index (ECMI) of patients using the R package glmnet 4.1.3, and the dependent variable of LASSO regression is patient survival days. SurvivalRandomForest with 1,000 trees was used to validate and rank the significance of 11 genes identified by LASSO regression in R version 3.6.4.

### Gene Set Enrichment Analysis

The gene set enrichment analysis (GSEA) algorithm assessed the biological processes that were enriched between different groups. The data in TCGA were first transformed in preparation for linear modeling using voom in R package limma 3.46.0. The differential genes between the two groups were calculated using the R package limma 3.46.0. Subsequently, they were preranked by log2 fold change and delivered to the R package clusterProfiler 3.18.1 for GSEA. Results with an adjusted p-value <0.05 were considered to be statistically significant.

### Annotation of the Tumor Microenvironment Cell Infiltration

To examine the immune cell infiltrating microenvironment, we quantified the enrichment levels of 64 immune signatures using the xCell algorithm (xCell: digitally portraying the tissue cellular heterogeneity landscape). We performed more thorough investigations using algorithms such as CIBERSORT (22), ssGSEA, quanTIseq, TIMER, and MCPcell in the R package immunedeconv version 2.0.4.

### Construction and Validation of A Neural Network Model

Differentially expressed genes (DEGs) between the two groups (PCR and RD) were assessed using the R package limma 3.46.0. The results were considered statistically significant when the p-value was <0.01. Using these DEGs, we established a neural network model using the neural net package in R 1.44.2. The number of neurons in the hidden layer was 15. We chose resilient backpropagation without weight backtracking as the core algorithm. The logistic function was set as the activate function. As stopping criteria, the threshold for the partial derivatives of the error function was 1*10-6.

### Statistical Analysis

We used independent t-tests and Mann–Whitney U tests to determine the statistical significance when comparing two groups with normally distributed and non-normally distributed variables, respectively. One-way analysis of variance (ANOVA) and Kruskal–Wallis tests were used to compare the differences between more than two groups (23). Spearman and distance correlational analyses were performed using the R package Hmisc 4.4.1. Objects with a coefficient >0.5 were considered strongly correlated (24). Cox regression analyses were performed to identify the prognostic factors. The OS and ECMI were determined using the R package survival, and cutoff values were determined before generating all survivorship curves with the R package survminer. The R package forest plot 2.01 was used to show the univariate prognostic analysis in different cohorts. Software Cytoscape v3.9.0 was used to construct the pathway networks. All of the heatmaps were plotted using the R package Complex Heatmap 2.4.3. OncoPrint was used to delineate the overview of gene mutation landscape, which was generated using the R package maftools 2.4.12. Confusion matrix tables were analyzed using the χ^2^ contingency test. The OS and risk scores were determined using the R package survival and cutoff values. Data comparisons were visualized using the R package ggplot2. OncoPrint was used to delineate the mutation landscape of TCGA using the maftools R package (25). All statistical analyses were two-sided and performed using R software. Statistical significance was defined as a p-value <0.05.

### Quantitative Real-time Reverse Transcription Polymerase Chain Reaction

From January 17, 2021, to September 19, 2021, patients with BC receiving neoadjuvant therapy were recruited in the Breast Department of Harbin Medical University Cancer Hospital. Patients were required to have a tumor size >2 cm and <5 cm before treatment and younger than 70 years (**Supplementary Table S16**). In total, 15 patients with pCR and 15 with RD were enrolled, and their baseline characteristics are shown in **Table 1**. BC tissue and tumor-adjacent tissue were obtained by core needle biopsy before the first cycle of neoadjuvant treatment. RNA was extracted using an RNeasy kit (Qiagen Sciences, Hilden, Germany), diluted using nuclease-free water, and electrophoresed on a denaturing formaldehyde agarose gel to visualize rRNA and ensure overall sample quality. RNA concentrations and purity were detected on an ultraviolet spectrophotometer (Harbin Medical University, Harbin, China). cDNA was obtained using a PrimeScript II 1st-strand cDNA synthesis kit (TaKaRa, Dalian, China). qPCR was performed using a LightCycler 480 real-time PCR machine (Roche) with SYBR. Glyceraldehyde-3-phosphate dehydrogenase (GAPDH) was used as the reference gene, and the relative gene expression was quantified using the cycle threshold (ΔΔCT) method. The primer sequences are listed in **Table 2**. This study was approved by the ethics committee of Harbin Medical University, and all patients provided a written informed consent.

**Table 1:** Baseline characteristics of the enrolled patients.

Characteristic	Cohort			P Value Between pCR and RD cohiorts
	All Patients (N=30)	pCR (N=15)	RD (N=15)	
Age, median (range), y	48.5 (28-66)	46 (28-66)	51 (31-65)	0.32
Pre long axis of tumor, median (range), cm	3.2 (2-5)	3.1 (2-5)	3.2 (2-4.7)	0.55
Pre short axis of lymph node, median (range), cm	1.1 (0-2.4)	1.3 (0.5-2.1)	0.8 (0-2.4)	0.26
Hormone receptor status, No. (%)				
Positive (>1%)	14 (47)	6 (40)	8 (53)	0.71
Negative	16 (53)	9 (60)	7 (46)	
Her2 status No. (%)				
Positive (IHC:3+/ISH:+)	20 (67)	12 (80)	8 (53)	0.25
Negative (IHC:1+/ISH:-)	10 (33)	3 (20)	7 (47)	

IHC, immunohistochemistry; ISH, in situ hybridization; pCR, pathologic complete response; RD, residule disease.

**Table 2:** Real-time qPCR primers.

Primer	Sequence (5′–3′)
OGN-qPCR-F	TCCTCTACTTGGACCATAATGC
OGN-qPCR-R	TGTAACTGGTGTCATTAGCCTT
GAPDH-qPCR-F	GAAGGTGAAGGTCGGAGTCA
GAPDH-qPCR-R	TTGAGGTCAATGAAGGGGTC

## Results

### Immune-Related Genes that Correlate With the Extracellular Matrix and Their Application to Identify the Extracellular Matrix Signature

A total of 11 BC cohorts (TCGA-BRCA, GSE58812, GSE16446, GSE48391, GSE69031, GSE32642, GSE50948, GSE66399, UCSC Caldas, UCSC Vijver, and Metabric-BRCA) were deemed suitable for our study. To investigate the ECM signature on the gene level, the “core matrisome” gene set was obtained from MatrisomeDB. This gene set consists of all ECM-related genes, including 195 ECM glycoproteins, 44 ECM collagens, and 35 proteoglycans (**Supplementary Table S1**). The GSVA score of this gene set was calculated to determine the level of ECM in each sample, and this value was designated as the ECM score (**Supplementary Table S2**).

**Figure 1:**
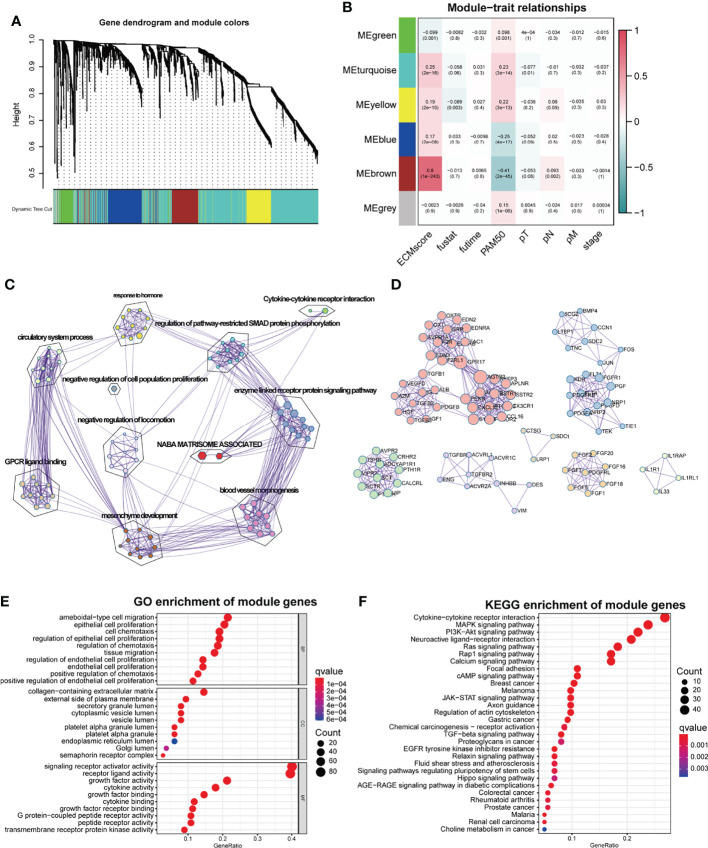
WGCNA for the ECM-related immune genes. **(A)** Cluster dendrogram generating gene modules. **(B)** Correlation analysis of modules and ECM score, and other clinical information. **(C)** Metascape for the functional annotation of the genes in the Brown module. **(D)** PPI network for significant protein–protein interactions among genes in the Brown module. **(E)** GO functional enrichment analysis of genes in the Brown module. **(F)** KEGG functional enrichment analysis of genes in the Brown module.

ECM remodeling has a significant impact on the development of an immunosuppressive TME (26), demonstrating that there is a strong link between the ECM and immunity; hence, we attempted to include ECM-specific immune genes into ECM-related genes. Two thousand four hundred eighty-three immune genes (**Supplementary Table S3**) were extracted from the public gene dataset (www.immport.org). We used WGCNA to identify the ECM-related immune genes. The scale-free topology fit index was set to 0.9 for scale-free network construction (**Supplementary Figures S1A, B**), and correspondingly, the best power value was 3. Six modules were identified using the clustering dendrogram (**Figure 1A**). The correlation coefficient between the Brown module and the ECM score was 0.8 (**Figure 1B**), suggesting that the Brown module is selectively expressed in samples with a high degree of the ECM component. In addition, this module has a strong correlation with the PAM50 subtype and pathologic N stage of BC, indicating its clinical significance. There were 213 genes in the Brown module (**Supplementary Table S4**), and only one gene (OGN) was shared between this module and the original ECM-related gene list.

To perform a comprehensive analysis of the genes in the Brown module, we used Metascape, an online omics data analysis portal, to functionally enrich these genes to GO, KEGG, Canonical pathways, Hallmark gene sets, etc. The top 20 enriched terms were listed (**Supplementary Figure S1A**), demonstrating that the Brown module genes were primarily enriched in Matrisome-associated signaling pathways and enzyme-linked receptor protein signaling pathways (**Figure 1C**). All protein–protein interactions (PPIs) among input genes were extracted from PPI data sources and used to construct a PPI network. The network was subjected to GO enrichment analysis to extract “biological” meanings. The MCODE algorithm was then used to identify neighborhoods where proteins were densely connected (**Figure 1D**). On the cellular component level, GO functional enrichment analysis revealed that these genes were enriched in pathways involving collagen-containing ECM and outer plasma membrane (**Figure 1E**, **Supplementary Figure S1C**). KEGG analysis revealed that the genes were enriched in the cytokine–receptor interaction and other pathways associated with cancer progression such as Mitogen-Activated Protein Kinase (MAPK) and Phosphoinositide-3-kinase (PI3K-Akt) pathways (**Figure 1F**).

In light of the significant role played by the Brown module in the development and biological process of BC, we merged the Brown module and genes from MatrisomeDB to form a new ECM-related gene list. To determine the potential value of the ECM-related genes, we first randomly divided TCGA data into training and testing groups in a 7:3 ratio (**Supplementary Tables S5, S6**). We attempted to make this combined ECM-related gene list as concise as possible to obtain a more exhaustive list of the ECM-related genes. Therefore, we performed univariate Cox regression in the training group to first filter out 477 survival-related genes with log-rank p < 0.01 (**Supplementary Table S7**). To create a more interpretable model and improve the prediction accuracy, LASSO regression was used to force the sum of the absolute value of the regression coefficients to be less than a fixed value. This forces coefficients of certain genes to zero, thereby excluding them from influencing predictions. Consequently, 11 prominent genes (TSLP, NOS2, SDC1, TPT1, PXDNL, TECTA, TNN, VWA5B2, ZP1, ZP2, and CHAD) with non-zero coefficients were identified. Four of these genes (PXDNL, TECTA, TNN, and VWA5B2) were in the original list of the ECM-related genes. These 11 genes were determined to score both training set and testing set samples using the following formula: -0.4127*TSLP + 0.2746*NOS2 + 0.1489*SDC1 + -0.0853*TPT1 + 0.1189*PXDNL + 0.9493*TECTA + -0.1793*TNN + 0.2093*VWA5B2 + 0.1448*ZP1 + 0.1773*ZP2 + -0.0698*CHAD. Among these 11 genes, the Brown module contributed to TSLP, NOS2, SDC1, and TPT1. The score was designated as the ECMI, and the 11 genes were designated as the ECMI genes.

**Figure 2:**
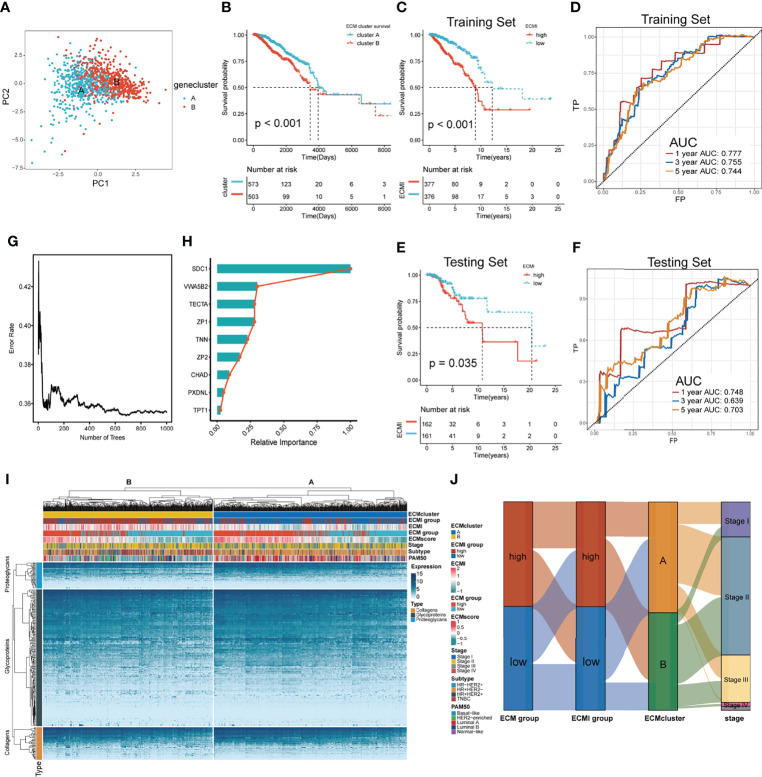
Identification of the ECM signature in TCGA. **(A)** Sample clustering by PCA in TCGA dataset. **(B)** Kaplan–Meier survival analysis of the two clusters in TCGA. **(C, D)** The Kaplan–Meier survival analysis and ROC curve analysis in the training sets, respectively. **(E, F)** The Kaplan–Meier survival analysis and ROC curve analysis in the testing sets, respectively. **(G)** The relation between the error rate and the number of trees in the process of running SurvivalRandomForest algorithm. **(H)** The relative importance of 9 of the genes consisting the ECMI. **(I)** Heatmap exhibiting unsupervised clustering of 11 prominent ECMI-related genes for patients in TCGA. Tumor stage, IHC subtype, ECM group, ECMI, ECM score, and PAM50 subtypes are shown as patient annotation. **(J)** Sanky plot showing the different ECM groups in the ECMI groups, ECM clusters, and tumor stages.

To further validate the clinical and transcriptomic characterization of the 11 ECMI genes consisting of ECMI, we clustered TCGA samples into ECM clusters A and B using an unsupervised clustering algorithm (**Supplementary Figures S1D–G**). The optimal number of clusters was evaluated using the ConsensusClusterPlus package (**Supplementary Figure S1F**). The clustering findings were most consistent when the number of clusters was set to two (K = 2) (**Supplementary Figures S1G, H**). Principal Components Analysis (PCA) differentiated the samples within TCGA dataset (**Figure 2A**). The delineated groups based on the 11 ECMI genes also confirmed a lower survival probability curve for cluster B (**Figure 2B**). Heatmaps (**Supplementary Figure S1I**) show the 11 gene expression patterns, of which CHAD (immunologically relevant component) and TNN (ECM macromolecular) levels were significantly higher in cluster A, whereas SDC1, an immune-related gene, was more abundant in cluster B.

Using a median cutoff, patients in the training group were stratified into low- and high-risk groups, and Kaplan−Meier (KM) plots were generated, as shown in **Figure 2C**. A more specific finding was that those in the group with reduced ECMI had significantly improved survival results. To further examine the efficacy of the ECMI, receiver operating characteristic (ROC) curve analyses were performed, yielding a 1-, 3-, and 5-year area under the curve (AUC) values of 0.777, 0.755, and 0.744, respectively (**Figure 2D**). Contrary to expectations, the AUC was similarly significant in the testing set, with 1-, 3-, and 5- year AUC values of 0.748, 0.639, and 0.703, respectively (**Figure 2E**), and the KM curve was also clearly separated in patients from the testing set, with a p-value <0.05 (**Figure 2F**). The findings demonstrated that ECMI was a reliable prognostic biomarker for predicting the 3- and 5-year survival status of BC patients. A SurvivalRandomForest algorithm was used to validate the LASSO regression results. The number of trees was initially set to 1,000 (**Supplementary Figure S2A**), and the lowest error rate was reached when the tree number was 687, at which point the absolute and relative importance of these genes was extracted (**Figures 2G, H**; **Supplementary Figure S2B**). The ECM group (separated by median GSVA score), ECMI group (separated by median ECMI), and ECM cluster were highly congruent, as measured by the sanky plot (**Figure 2J**).

### Clinical and Biological Value of the Extracellular Matrix Index

**Figure 3:**
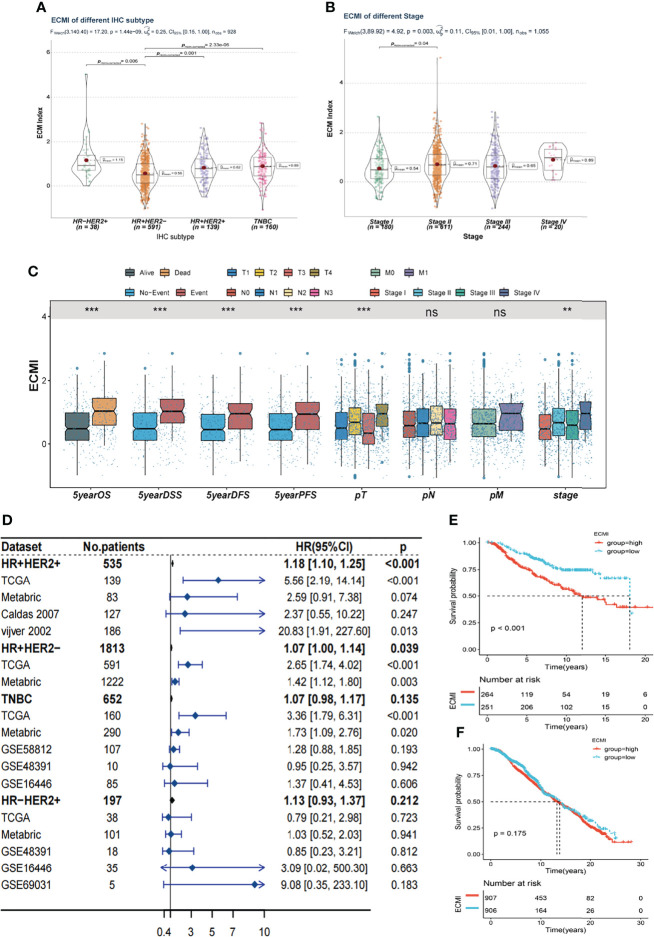
Clinical value of the ECMI. **(A)** The ECMI levels in four different IHC subtypes in TCGA. **(B)** The ECMI levels in four different clinical stages in TCGA. **(C)** Boxplot of the ECM score in different clinical subgroups in TCGA. *p < 0.05, ***p < 0.001, ****p < 0.0001. ns, not statistically significant. **(D)** Forestplot was plotted to present the predictive value of the ECMI in each subtype and cohort. **(E)** Kaplan–Meier survival analysis of the ECMI high group and ECMI low group in merged HR+HER2+ cohort. **(F)** Kaplan–Meier survival analysis of the ECMI high group and ECMI low group patients in merged HR+HER2- cohort.

Tumors belonging to the Luminal-A PAM50 subtype have a relatively lower ECMI than those of Basal-like, HER2-enriched, and Luminal B tumors (**Supplementary Figure S3A**). Similarly, the IHC molecular subtypes showed that the HR+HER2- subtype had the lowest ECMI (**Figure 3A**). These findings demonstrate that the ECM composition of HR+ BC differs from that found in previous studies (27). This has previously been demonstrated by other studies. In addition, patients in TCGA with a high ECMI had a high risk for all subtypes except HR-HER2+ (**Supplementary Figures S3B–E**). However, the American Joint Committee on Cancer (AJCC) stage did not indicate a significant difference in ECMI; there was only a marginally significant difference between patients in stage II and stage I (**Figure 3B**). In TCGA cohort, patients with more event occurrences of 5-year OS, disease-specific survival (DSS), disease-free survival (DFS), and progression-free survival (PFS) had a significantly higher ECMI, although the pathologic N stage (pN) and the presence of the metastatic disease did not correlate with the ECMI (**Figure 3C**).

To better validate the efficacy of the ECMI, we performed an external validation on 7 additional BC cohorts. Initially, we eliminated the batch effect for these eight cohorts, including TCGA group (**Supplementary Table S8**). Upon dividing the specific datasets by the best cutoff value of the ECMI, significant differences in OS were observed between the ECMI low and high groups for all BC datasets except for the UCSC Caldas set and GSE69031 (**Supplementary Figures S3F–J**). Because BC has robust heterogeneity between different IHC subtypes, we performed subtype analysis (**Supplementary Figures S4A–D**) and deleted data resulting in excessive heterogeneity before merging data from different datasets. A forest plot (**Figure 3D**) was generated to illustrate the predictive value of ECMI in each subtype and cohort. The hazard ratio (HR) was statistically significant in HR+HER2+ (HR = 1.18, p < 0.001) and HR+HER2-, demonstrating that the ECMI is a reliable risk factor in BC when both hormone receptor and HER2 are positive. However, there was no statistical significance in the Triple Negative Breast Cancer (TNBC) and HR-HER2+ subtypes, indicating that the hormone receptor is essential for the ECM to perform its function. The survival curve showed the risk effect of the ECMI in the HR+/HER2+ group (**Figure 3E**). In contrast, there were no significant differences between the ECMI high and low groups in other subtypes (**Figure 3F**; **Supplementary Figures S3E, F**). The significant difference in the HR+/HER2+ group may also show a robust association between HER2 and the ECM. For example, in the study by Hanker et al. (28), the ECM component was associated with a significantly inferior clinical response to neoadjuvant anti-HER2 therapy in HER2+ BC patients. Collectively, these findings indicated that the ECMI may be a significant predictive factor in BC, particularly in the HR+/HER2+ subtype.

**Figure 4:**
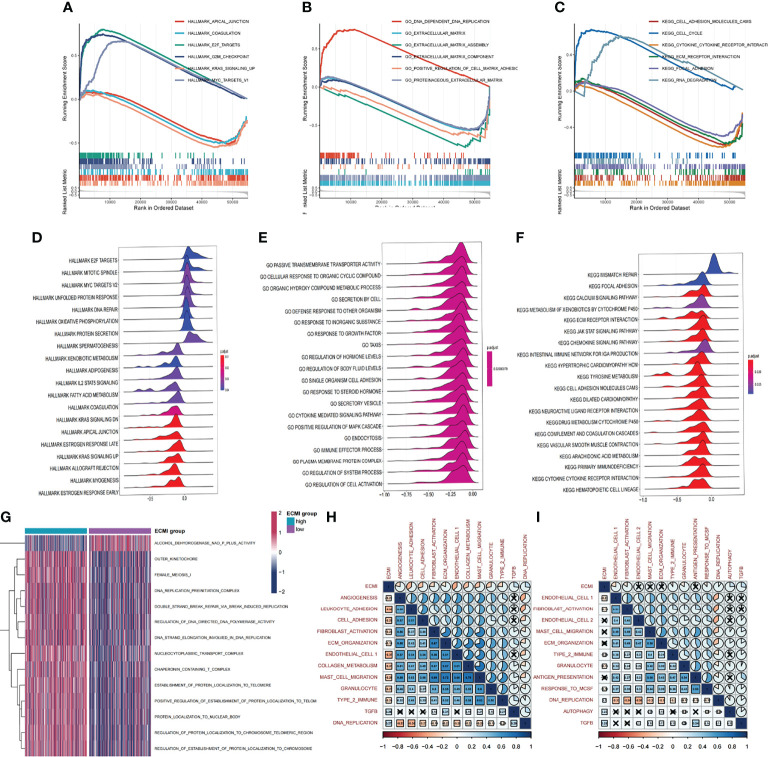
Biological value of the ECMI. **(A–C)** GSEA running plot showing the biological process of Hallmark, GO, and KEGG enriched in ECM high and low. **(D–F)** Ridge plots showing the top enriched biological process of GO, KEGG, and Hallmark in TCGA. **(G)** GSVA exhibiting the different expressed gene sets between the two groups. **(H, I)** The correlation between biological process and the ECMI in both TCGA and Metabric cohorts (the row annotation and column annotation are the abbreviation of the gene sets).

**Figure 5:**
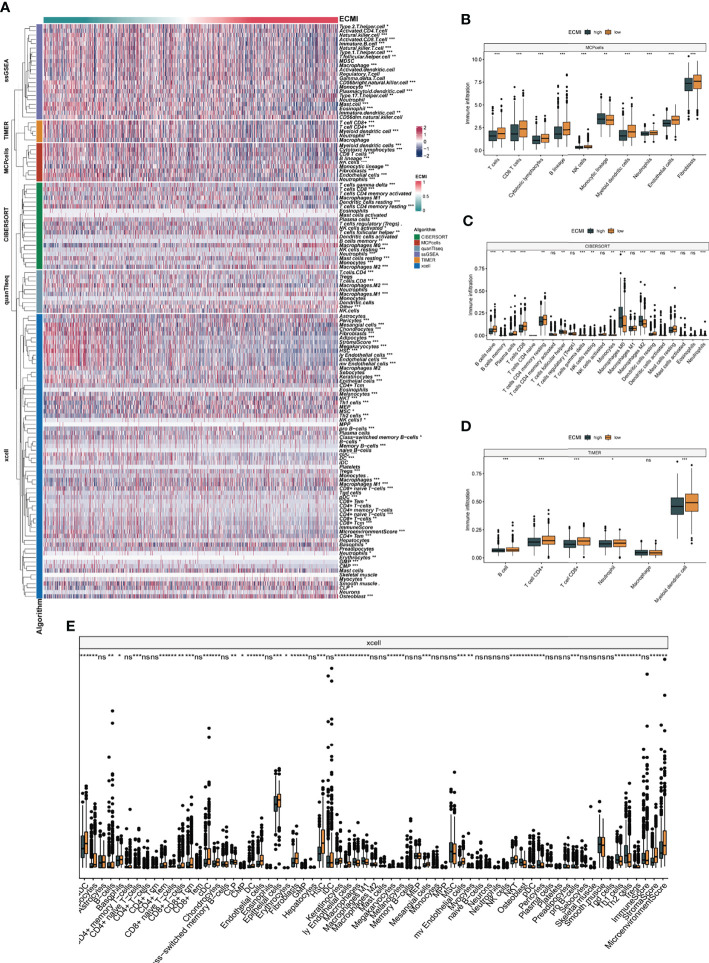
The landscape of immune infiltration based on the ECMI. **(A)** Heatmap for the infiltration of immune cells with row representing different kinds of immune cells and column representing different samples with the ECMI increasing from left to right. **(B–E)** Box plots showing the immune infiltration levels in ECM high and ECMI low based on MCPcells, CIBERSORT, TIMER, and xCell algorithm. *p < 0.05, ***p < 0.001, ****p < 0.0001. ns, not statistically significant.

We aimed to gain a deeper understanding of the relationship between ECM phenotypes and key biological processes, particularly in light of the remarkable performance of the ECMI on the clinical outcomes of BC patients. GSEA was performed to determine the most highly enriched gene set in the GO gene set, KEGG gene set, and Hallmark gene set (**Supplementary Tables S9–S11**). The top 20 enriched gene sets with adjusted p-value <0.001 in GO, KEGG, and Hallmark were listed (**Figures 4A–C**), and the ridge plots (**Figures 4D–F**) and bubble plots (**Supplementary Figures S5A, B**) were plotted to indicate the significant degree of each pathway in the three gene sets. The findings revealed that the ECMI negatively correlated with ECM assembly and ECM component processes and positively correlated with processes involved in DNA replication and DNA repair (**Figure 4B**). Moreover, in the hallmark gene set, the ECMI was positively related to estrogen response early and late process, confirming our previous hypothesis that the ECM composition is more active in HR+ BC (**Figure 4A**). To further compare the different biological characteristics between the ECMI low and high groups, we performed GSVA to extract the DEG set. The top 14 significant pathways are shown in **Figure 4G**, and we found that most of the processes were involved in the cell cycle and DNA replication. We also attempted to determine if a correlation existed between the ECM-relevant gene signatures and the ECMI. We found that the ECMI correlated negatively with blood vessel endothelial cell proliferation, collagen metabolism, Transforming Growth Factor-β (TGF-β) production, and angiogenesis but positively with processes involved in DNA replication and leukocyte adhesion (**Figure 4H**). The Metabric cohort also confirmed the validity of this finding (**Figure 4I**). Due to the strong association between the TME and the ECM (29), we analyzed the immune infiltration pattern of the ECM phenotypes using 6 different algorithms (**Figure 5A**; **Supplementary Table S12**). In total, a higher ECMI showed a scarce TME with significantly low stromal score and microenvironment score, and the relation between ECMI and immune cells and the relationship between immune cells were presented under the algorithm of xCell (**Supplementary Figure S3C**). However, the monocytic lineage cells were found to be more abundant in the ECMI high group based on MCP, CIBERSORT, and xCell algorithms (**Figures 5B, C, E**). Specifically, CIBERSORT and xCell exhibited a higher M0 in the ECMI high group. In contrast, the ECMI high group exhibited a higher M1 in CIBERSORT but a higher M2 in xCell, a finding that will require further study to validate. Surprisingly, CD8+ T cells were enriched in the ECM low group in all six algorithms, indicating an antitumor TME that could improve the long-term survival of patients (**Figures 5B–E**; **Supplementary Figures S5D, E**).

### Multi-Omics Features of The Extracellular Matrix Phenotypes in TCGA Cohort

**Figure 6:**
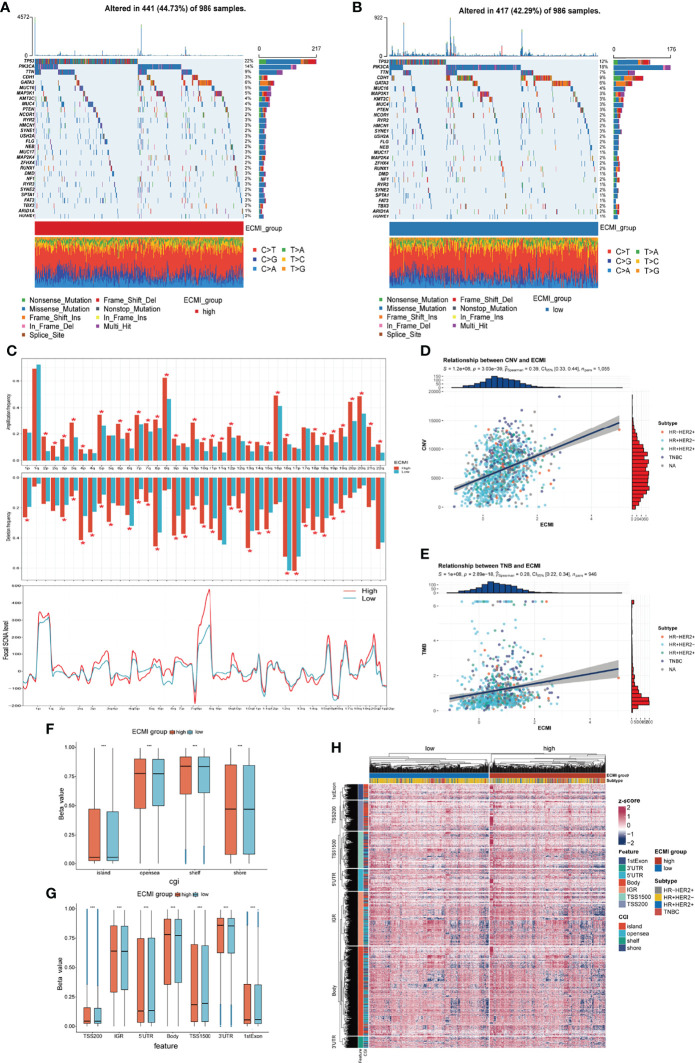
Genomic features of the ECMI high and low groups. **(A, B)** List of the most frequently mutated genes in ECMI high and low groups. **(C)** Global distribution of gain- or loss-of-function mutation in the 22 human chromosomes in the two groups. Amplification of genes is marked in red. Deletion of genes is marked in turquoise. The above diagram represents the arm-level CNV, and the bottom represents the focal-level CNV. **(D, E)** Correlation between the ECMI and total CNV or TMB in TCGA. **(F, G)** Methylation difference between the ECMI high and low groups in different CGI coordinates and feature types. **(H)** Heatmap exhibiting the DNA methylation pattern of the ECMI high and ECMI low groups. The location of each DNA methylation site is annotated on the left. The IHC subtype is shown in the top annotation.

Somatic variation analysis (SVA) and CNV were performed in TCGA dataset to investigate the genomic characteristics of the ECMI high and low groups. According to SVA, mutations in TP53 (22%), PI3KCA (14%), TTN (9%), and GATA3 (6%) were most highly enriched in the ECMI high group (**Figure 6A**). In contrast, PI3KCA (18%), TP53 (12%), CDH1 (9%), and TTN (7%) mutations were enriched in the ECMI low group (**Figure 6B**). TP53 mutations are more prevalent in the ECMI high group, whereas PI3KCA mutations are more prevalent in the ECMI low group. All of these genes, except for CDH1, had missense mutations as the predominant gene mutation type, with frameshifting deletion being more prevalent in the ECMI high group and frameshifting insertion being more common in the ECMI low group.

A global CNV and focal Somatic Copy Number Alterations (SCNA) level profile was generated by comparing the two groups (**Figure 6C**), which revealed a significant difference in both arm and focal levels. The total CNV was also positively correlated with the ECMI (**Figure 6D**; Spearman correlation, r = 0.39, p = 3.03e−39), and the correlation was more predominant in the HR+/HER2+ subtype (**Supplementary Figures S6A–D**; Spearman correlation, r = 0.42, p = 3.15e−07) and insignificant in the HR-/HER2+ group (p = 0.811).

Similarly, the tumor mutational burden (TMB) is correlated with immune recognition and clearance, immune checkpoint inhibitor response (30, 31), and chemotherapeutic drug function. Examining the TMB is therefore necessary for the accurate selection of systemic therapy (30, 31). We observed a positive correlation between the TMB and the ECMI (**Figure 6E**; Spearman correlation, r = 0.28, p = 2.89e−18). While different IHC subtypes demonstrate varying degrees of correlation between the ECMI and the TMB (**Supplementary Figures S6E–H**), the highest correlation was observed in the TNBC group (Spearman correlation, r = 0.3, p = 3.27e−04).

Epigenetics has long been recognized as a vital element contributing to tumor maintenance and in the development of cancer’s well-known characteristics. Epigenetic abnormalities have long been identified as an essential factor contributing to tumorigenesis and immune surveillance among all tumor types. Therefore, we attempted to establish the relationship between ECMI and DNA methylation. The ECMI high group had a higher level of DNA methylation in nearly all DNA sites except for the 5′ untranslated region, TS1500, and the 1st Exon, which is associated with inhibiting mRNA translation, which was enriched in the ECMI low group (**Figures 6F–H**).

### Extracellular Matrix Signature for Predicting Hormone Therapy and Neoadjuvant Treatment Benefits

**Figure 7:**
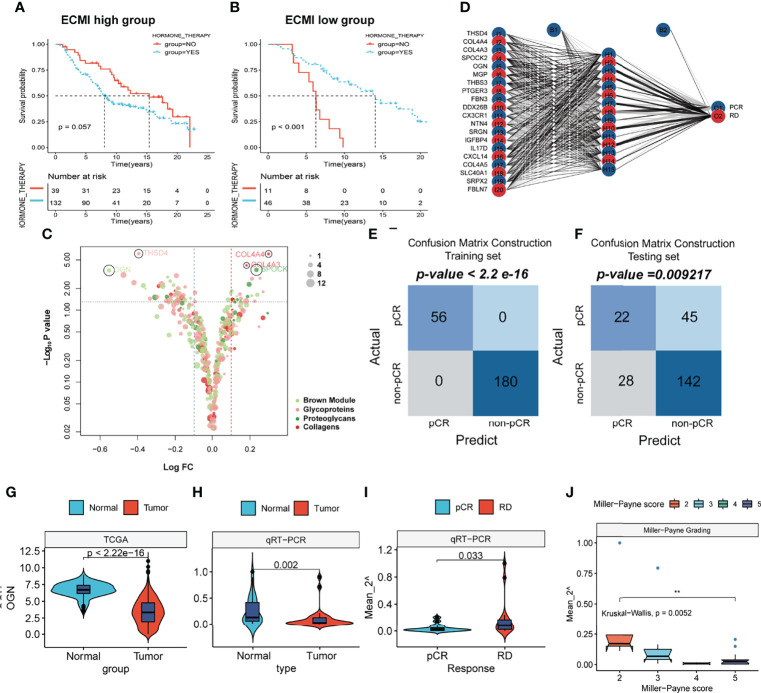
The prediction of hormone and neoadjuvant chemotherapeutic efficacy based on the ECM signature. **(A, B)** Kaplan–Meier survival analysis of the patients with or without hormone therapy in ECMI high and low groups. **(C)** Volcano plot showing the differentially expressed genes (DEGs) between patients with pCR and non-pCR. **(D)** Schematic diagram of the neural network established by the 20 DEGs. **(E, F)** Contingency tables show the consistency between the predicted clusters and the actual clusters. **(G, H)** Violin plots showing the different levels of OGN between Normal and Tumor tissues in TCGA cohort and Harbin Medical University cohort. **(I)** Violin plots exhibiting the different levels of OGN between pCR and RD tissues. **(J)** Violin plots exhibiting the different levels of OGN among different MP scores.

To improve survival outcomes for BC patients, it is critical to use a systematic approach that includes adjuvant and neoadjuvant chemotherapy as well as hormone therapy. Currently, there is no biomarker that can accurately predict the benefits of each of these systemic treatments and further guide the selection of patients who can benefit the most. Given the efficacy of the ECM-related genes in predicting the survival outcomes for HR+HER2+ patients, we assessed their performance in predicting the therapeutic effects of hormone therapy and NAC. The Metabric cohort was used in this study. First, patients who were hormone receptor-positive and HER2+ and had received hormone therapy but no chemotherapy were assigned into the ECMI low and high groups by the best cutoff value based on their OS time. The same cutoff value was also used to separate patients who had not received both hormone therapy and chemotherapy. After combining group information and the history of hormone therapy, the Metabric cohort was further divided into Hormone+/ECMI high, Hormone−/ECMI high, Hormone+/ECMI low, and Hormone−/ECMI low groups. Surprisingly, there were no marked benefits from hormone therapy for patients in the ECMI high groups probably due to more risk factors for these patients (**Figure 7A**; p = 0.205). However, when ECMI was low, there were significant differences between the hormone therapy and non-hormone therapy groups (**Figure 7B**; p < 0.01), indicating better hormone therapy responses than those in patients with a high ECMI.

NAC is also a critical strategy for patients. It is aimed at increasing the likelihood of tumor control *via* rapid assessment of drug efficacy and can accelerate the development and approval of treatments for early BC (32). The indicator for the therapeutic effects of NAC is a pathologic complete response (pCR), which is considerably associated with long-term clinical benefits in BC (33). To investigate the value of the ECM-related genes in predicting responses to neoadjuvants, we selected 4 datasets (GSE16446, GSE32642, GSE50948, and GSE66399) containing pCR information in GEO and excluded the batch effects from analysis. The total number of patients were assigned into two groups based on whether or not they had their pCR data. Next, we identified the DEGs from the ECM-related gene list (**Supplementary Table S13**, p < 0.01, log FC >0). In total, 20 DEGs were extracted, with THSD4, COL4A4, COL4A3, SPOCK2, and OGN being the top 5 significant genes (**Figure 7C**). To predict the pCR, patients were randomized into training and testing cohorts. Then, the neural network carried out by resilient backpropagation with weight backtracking was applied to establish the model (**Figure 7D**). The hidden layer contained 15 neurons, the threshold was set to 0.000001, logistic regression was chosen as the activated function, and Scatter diagrams plotted the generalized weights for the 5 prominent genes (**Supplementary Figures S7A–E**). The training group exhibited a notable stratification with 100% accuracy in both specificity and sensitivity, as revealed by confusion matrix construction (**Figure 7E**; **Supplementary Table S14**). In the testing group, due to the low rate of pCR (only about 20%) in BC patients, we focused on the positive predictive value (44%) and negative predictive value (75%), which were acceptable (**Figure 7F**; **Supplementary Table S15**). Thus, our model can be used in conjunction with other clinical factors to identify patients who can benefit the most from neoadjuvant treatment to receive treatment before surgery and perform surgery first for those resistant to chemotherapy.

This result was validated in a cohort obtained from January 17, 2021, to September 19, 2021. Since OGN has the foremost predictive significance and is also the representative gene of the new ECM-related gene list, we performed qRT-PCR to assess OGN expressions in each sample (**Supplementary Table S17**). Compared to normal tissues, OGN was significantly suppressed in BC tissues from both TCGA and our cohorts (**Figures 7G, H**). Patients with pCRs to neoadjuvants exhibited significantly low levels of OGN compared to those with residual disease (RD) (**Figure 7I**). Furthermore, we used Miller–Payne (MP) grading, which is a more accurate evaluation system to assess responses to NAC. There were significant differences in OGN expressions between MP: 2 and MP: 5 groups (**Figure 7J**), implying that OGN has a marked effect in inhibiting drug delivery.

Additionally, we determined whether the ECMI can be used to predict the success of immunotherapy treatments. In the IMgivor 210 cohort, we did not find any differences in the ECMI between patients achieving Partial Response (PR), Stable Disease (SD), Complete Response (CR), or Progressive Disease (PD) (**Supplementary Figure S7F**). These findings provide compelling evidence that the ECMI and the neural model based on our ECM-related gene list predict poor prognosis and responses to hormone therapy and NAC.

## Discussion

As a major component of the TME (5, 34), the abnormal composition of the ECM mediates (35) all of the cellular processes involved in cancer progression, migration, and invasion. In BC, Wishart et al. (36) found that the ECM scaffold extracted from tissues after decellularization has a significant value in studying the biological behaviors of TNBC. Some of the genes with the ability for comodulating the ECM-associated matrisome networks, like BRD4, are oncogenic but not tumor-suppressive in TNBC, implying that targeting the ECM or its network may be a viable method for disease treatment. However, the ECM is heterogeneous among diverse tumor types, and interactions between the ECM and another component of the TME are a mystery. For instance, in gastric cancer (GC), the stiffness of the ECM interplays with DNA methylation of promoter regions of the mechanosensitive YAP, and this epigenetic regulation of the biophysical properties of the ECM of GC may be a potential therapeutic strategy for cancer progression inhibition (37). In contrast, in pancreatic ductal adenocarcinoma, the ECM is a protective factor. Impairment of the ECM with an anti-LOXL2 antibody *in vivo* facilitated tumor progression and lowered the OS outcomes (38). In small-cell lung cancer (SCLC) (39), based on differentially expressed ECM proteins in patient samples with non-small cell lung carcinoma, a five-gene prognostic signature was generated with independent prognostic value to identify patients in need of further adjuvant therapy after surgical resection.

To date, depiction of the ECM in most studies is based on the ECM component-related genes, regardless of other immunomodulating factors that could have a great impact on the ECM. The potential efficacy of the ECM in predicting the prognosis and systemic treatment outcomes has not been investigated. We used WGCNA to assess the overall ECM condition by evaluating the ECM-related immune genes. Through LASSO regression and machine learning, we shortened the gene list to facilitate the ECM composition pattern analysis and generated the ECMI. Then, we used the ECMI to depict the comprehensive landscape of clinical and multi-omics traits of the ECM in BC. In addition, the ECMI was used to predict long-term survival and systematic therapy benefits in BC. In general, the ECMI is a decisive risk factor in different IHC subtypes of BC and other datasets.

Findings from TCGA were validated by the Metabric cohort and UCSC Vijver. In the Metabric cohort, hormone therapy exhibited inferior effects in those with high ECMI. In the cohort merged by GSE16446, GSE32642, GSE50948, and GSE66399, we identified DEGs from the ECM-related gene list between pCR and RD groups. These DEGs exhibited better performance in predicting responses to neoadjuvant therapy. Previous findings found that the ECM has a substantial influence on TNBC. In this study, the ECMI performed well in predicting survival outcomes, particularly for HR+/HER2+ BC patients. GSEA revealed positive correlations between the ECMI and the biological process: estrogen response. The correlation between the CNV and the TMB was more significant in HR+/HER2+ BC than that in the other subtypes. Thus, further studies should be performed if there is an interplay between the ECM component and HR or HER2.

Angiogenesis and vessel endothelial cell proliferation are two biological processes that are highly correlated with the ECMI in both TCGA and Metabric cohorts. ECM deposition enhances angiogenesis and antiangiogenic therapy resistance (40). This could be because gradients of soluble Vascular Endothelial Growth Factor A (VEGFA) induce the generation of some specific active endothelial cells, which resolve the surrounding ECM and cause the growth of new vascular sprouts toward VEGFA (41). Therefore, the ECMI can be used as a biomarker to identify patients who might receive antiangiogenic therapies, such as bevacizumab.

Our results showed that BCs with a higher ECMI exhibited markedly higher levels of gene enrichment in DNA replication and a high level of mismatch repair and homologous recombination, which might be attributed to protection of the rapidly proliferating BC cells by the ECM. We hypothesized that the high DNA repair capacity might limit the efficiency of chemotherapeutic medications because chemotherapy causes DNA damage in rapidly proliferating cancer cells (42). Combined with close correlations between the ECMI with CNV and TMB, the capacity of our ECM gene list to predict systemic therapy benefits can be established.

Macrophages can be polarized into two distinct phenotypes: pro-inflammatory M1 and protumor M2. In the study by Witherel et al. (43), hybrid M1/M2 macrophage-conditioned medium controlled the ECM formation by generating a matrix with thicker and less aligned fibers. In comparison, M2 macrophage-conditioned media resulted in the formation of a more aligned matrix with thinner fibers. Therefore, altering the M1/M2 balance toward M2 may induce architectural and constitutional changes in the ECM with enhanced potential for downstream remodeling (43). In this study, we used six different algorithms to assess immune cell infiltrations. Interestingly, CIBERSORT and xCell exhibited a higher M0 in the ECMI high group, while the ECMI low group exhibited a higher M1 in CIBERSORT but a higher M2 in xCell. Low stromal and microenvironment scores were found in the ECMI high group, which helped us evaluate the TME status.

Given the vital role of epigenetics in regulating cancer progression and drug resistance (44–46), we investigated if the ECM features may change the epigenetic state of breast tumors and hence influence medication sensitivity. According to our findings, the ECMI high group had a greater variety of methylation sites.

In conclusion, we identified an ECM gene expression signature (ECMI) consisting of 11 ECM-related genes and established its prognostic value in BC. We comprehensively studied the landscape of clinical, biological, and multi-omics traits of the ECM compositional patterns in BC. A higher ECMI was closely correlated with the constitution of TME cells, angiogenesis, DNA replication, and IHC molecular subtypes, notably higher somatic mutation rates, and higher levels of DNA methylation and CNVs. Moreover, the ECMI was established to be a robust prognostic indicator and a predictive factor for benefits of hormone therapy. Lastly, OGN was extracted as the foremost valuable gene in our new ECM gene list by machine learning, and its predictive value for neoadjuvant treatment was validated by qRT-PCR. Establishment of the ECMI and neuron network based on DEGs will inform the application of suitable hormone therapy and NAC and form the basis for the development of innovative therapeutic approaches.

This study has some limitations. One important drawback was the absence of external real-world RNA-seq data that might be used to corroborate and verify our findings. Another limitation was that in-depth mechanisms such as the regulation of the processes of angiogenesis, cell cycle, and DNA replication were undetermined, and further tests should be performed to confirm these findings. Moreover, single-cell sequencing should be performed to assess the relationships between the ECM and alternation of the TME.

## Data Availability Statement

The datasets presented in this study can be found in online repositories. The names of the repository/repositories and accession number(s) can be found in the article/**Supplementary Material**.

## Ethics Statement

The studies involving human participants were reviewed and approved by the ethics committee of Harbin Medical University. The patients/participants provided their written informed consent to participate in this study.

## Author Contributions

JL conceived the project. BL wrote the manuscript. XY was responsible for statistical tests and data collection. YL and YD contributed to data analysis and interpretation. ZL and TL conducted the experiments. LY revised the manuscript and communicated with the journal and editorial office. All authors contributed to the article and approved the submitted version.

## Funding

This study was supported by Haiyan Foundation of Harbin Medical University (JJMS2021-15) and National Natural Science Foundation of China (Nos. 82072903).

## Conflict of Interest

The authors declare that the research was conducted in the absence of any commercial or financial relationships that could be construed as a potential conflict of interest.

## Publisher’s Note

All claims expressed in this article are solely those of the authors and do not necessarily represent those of their affiliated organizations, or those of the publisher, the editors and the reviewers. Any product that may be evaluated in this article, or claim that may be made by its manufacturer, is not guaranteed or endorsed by the publisher.

## Abbreviations

BC, breast cancer; ECM, extracellular matrix; DEGs, differentially expressed genes; pCR, pathologic complete response; RD, residual disease; SLNB, sentinel lymph node biopsy; IHC, immunohistochemistry; TCGA, The Cancer Genome Atlas; GEO, Gene Expression Omnibus; UCSC, University of California Santa Cruz; TPM, transcripts per kilobase million; GO, Gene Ontology; KEGG, Kyoto Encyclopedia of Genes and Genomes; SVA, somatic variation analysis; CNV, copy number variation; TMB, tumor mutational burden; CDF, cumulative distribution function; ANOVA, one-way analysis of variance; KM, Kaplan−Meier; ROC, receiver operating characteristic; ECMI, extracellular matrix index; AUC, area under the curve; GSEA, gene set enrichment analysis; GSVA, gene set variation analysis; OS, overall survival; DFS, disease-free survival; DSS, disease-specific survival; PFS, progression-free survival; pT, pathologic T stage; pN, pathologic N stage; pM, pathologic M stage; NAC, neoadjuvant chemotherapy.
