# Supplementary figures and images for: Combining Immune-Related Genes For Delineating the Extracellular Matrix and Predicting Hormone Therapy and Neoadjuvant Chemotherapy Benefits In Breast Cancer
Source: Front Immunol. 2022 Jul 14;13:888339. doi: 10.3389/fimmu.2022.888339 (PMC9331652; doi:10.3389/fimmu.2022.888339)

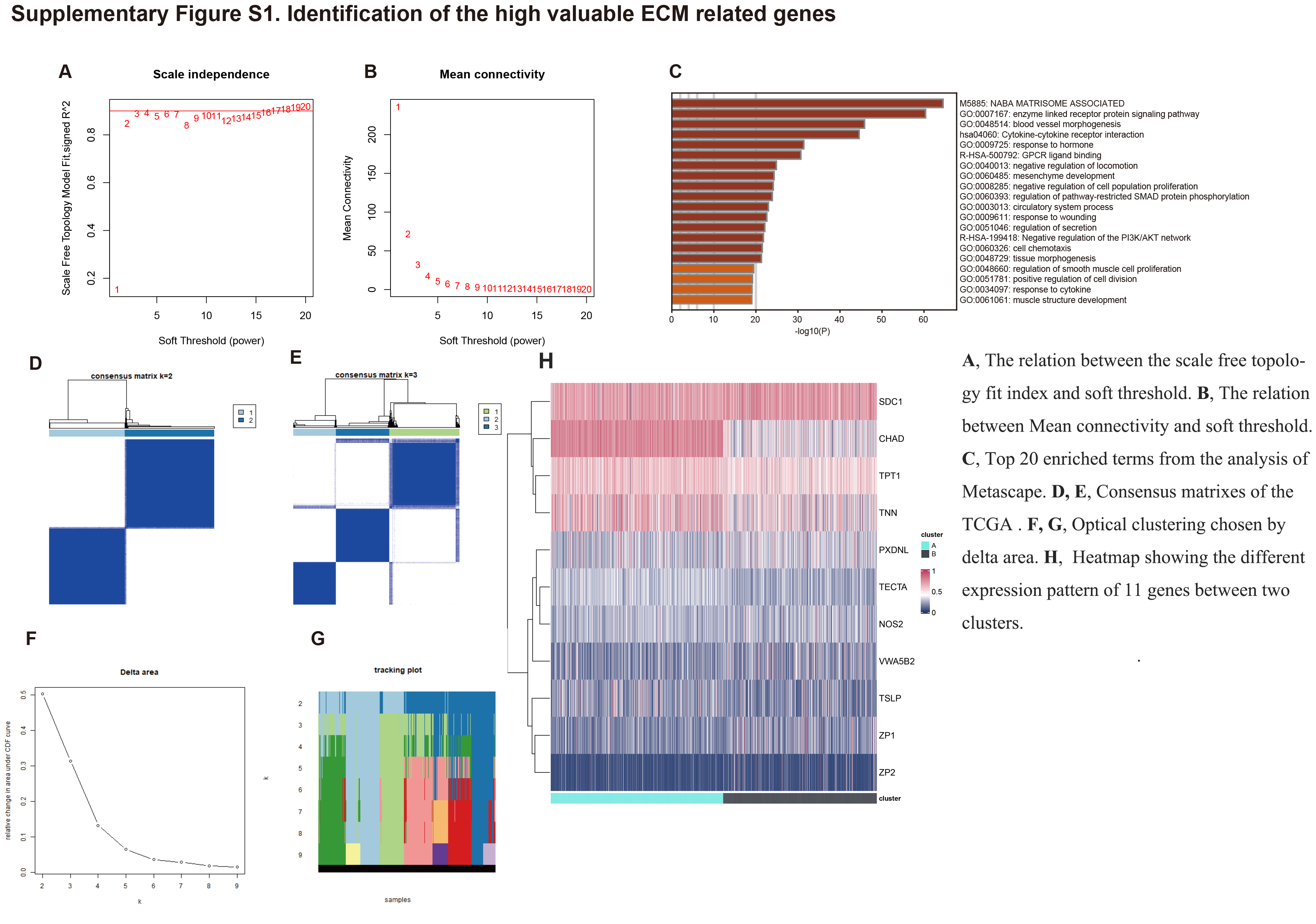

Supplement: Supplementary file 2 [file Image_1.tif]

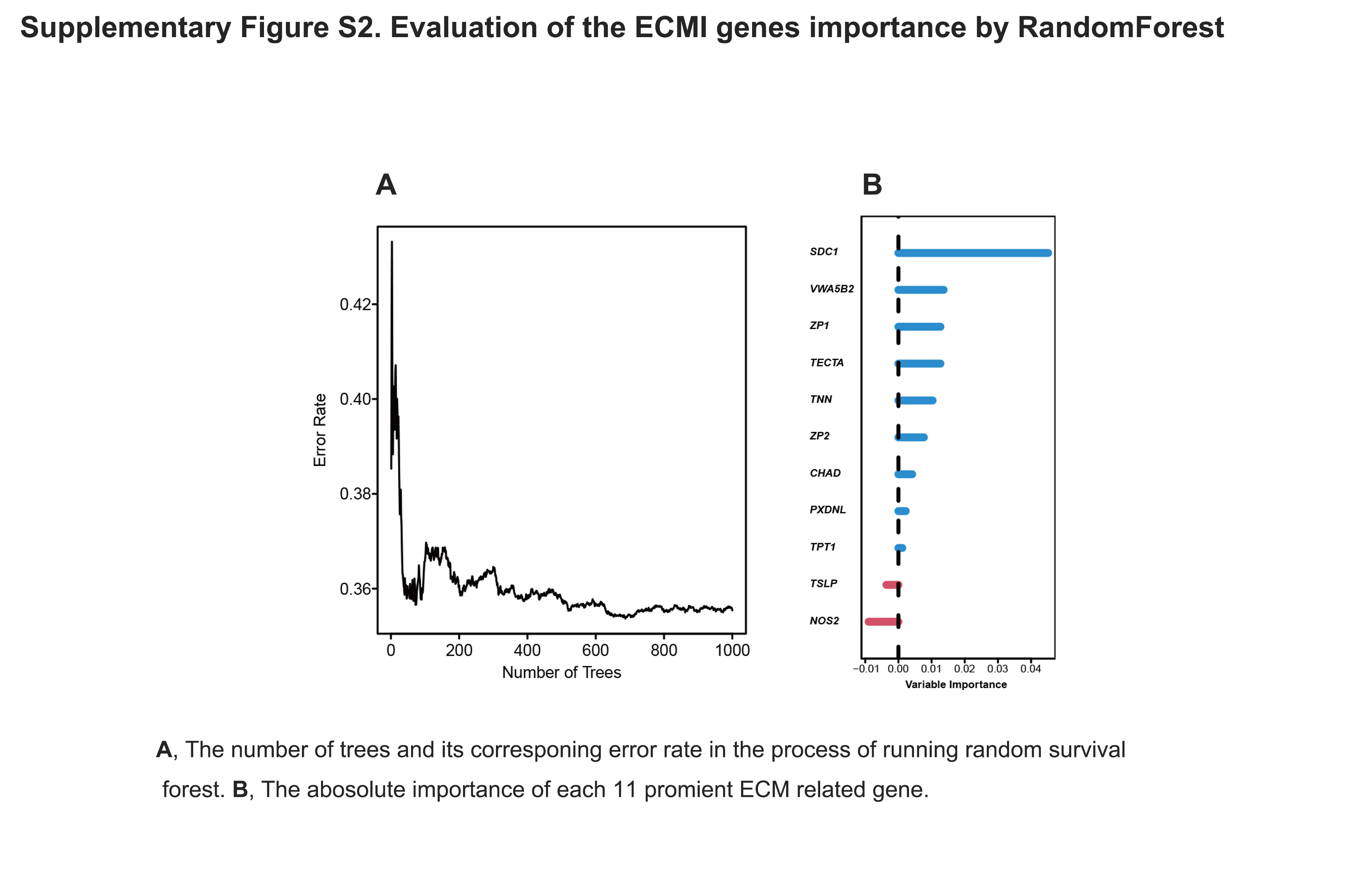

Supplement: Supplementary file 3 [file Image_2.tif]

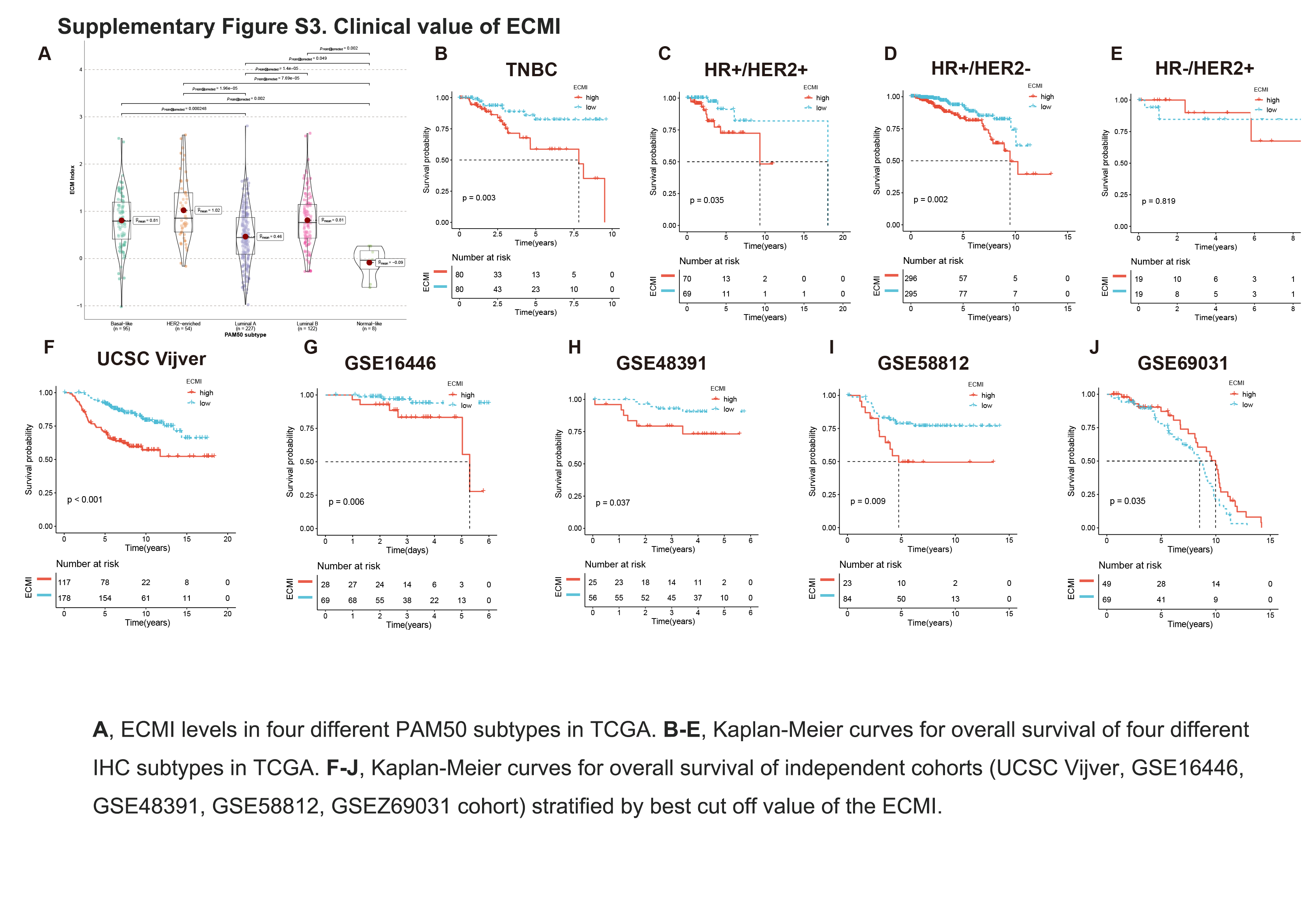

Supplement: Supplementary file 4 [file Image_3.tif]

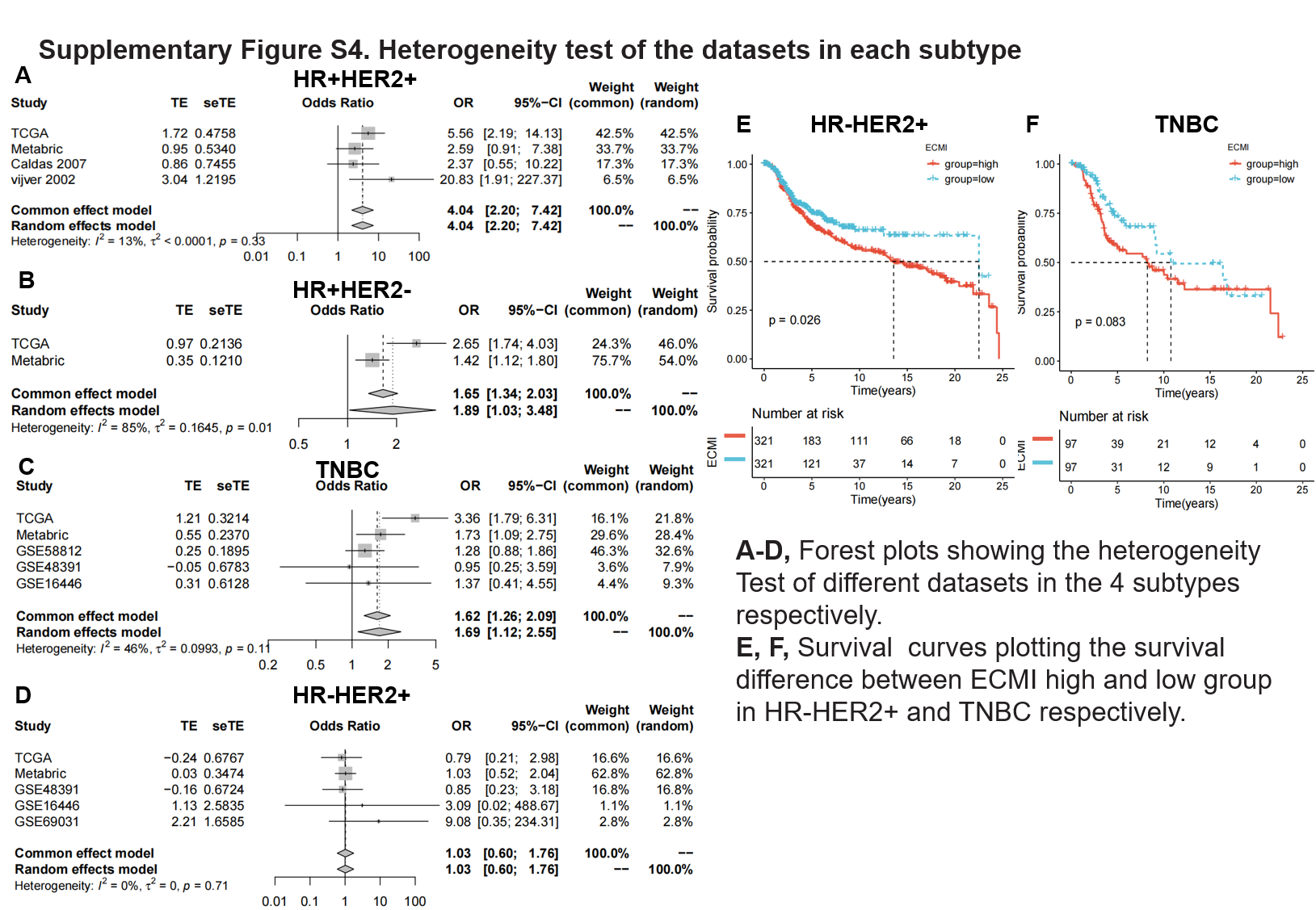

Supplement: Supplementary file 5 [file Image_4.tif]

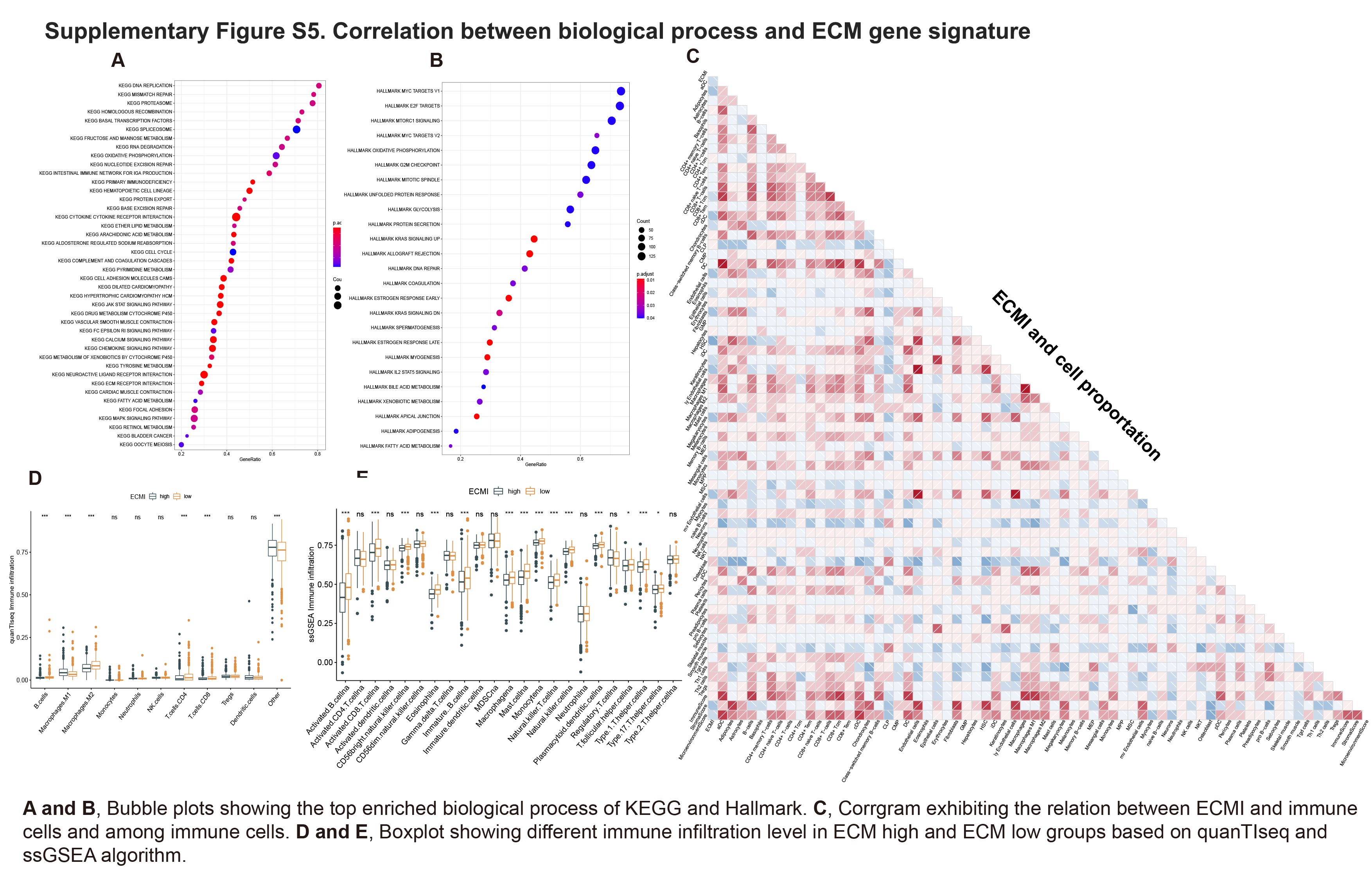

Supplement: Supplementary file 6 [file Image_5.tif]

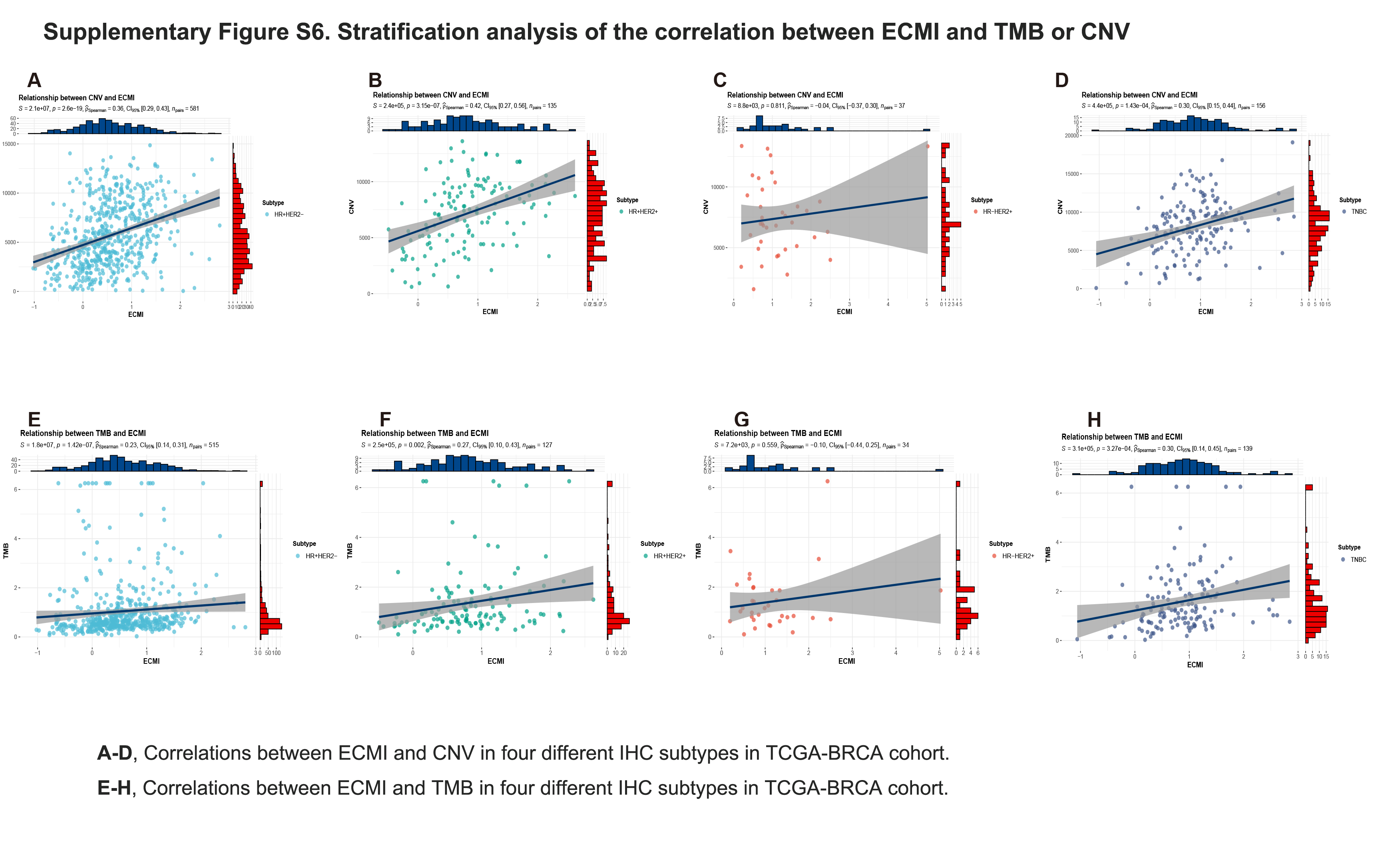

Supplement: Supplementary file 7 [file Image_6.tif]

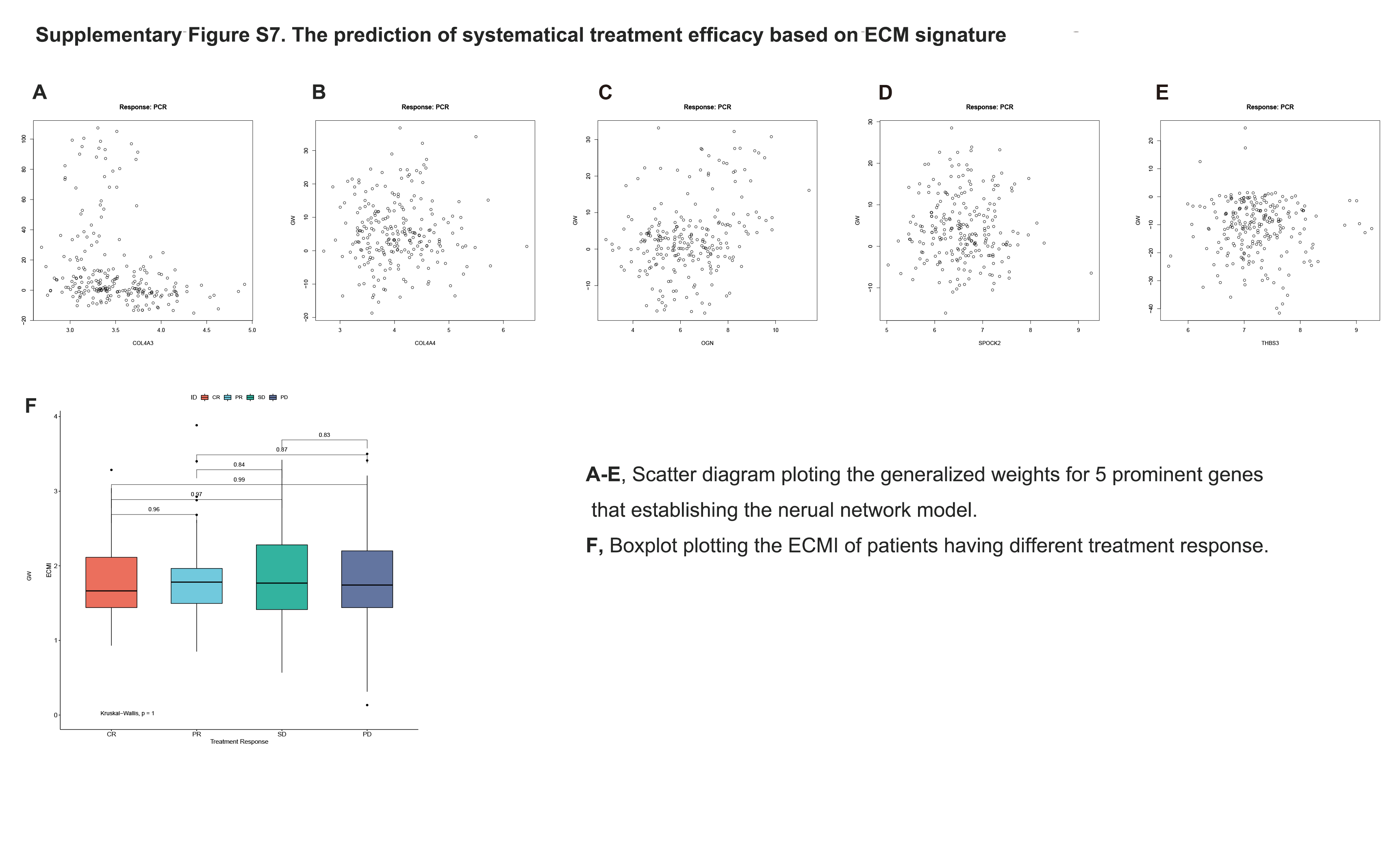

Supplement: Supplementary file 8 [file Image_7.tif]
